# Increasing fibroblasts and gingival collagen density in periodontitis rats by using cassava leaf extract

**DOI:** 10.1016/j.jtumed.2023.05.006

**Published:** 2023-05-19

**Authors:** Amandia D.P. Shita, Agustin W.S. Dharmayanti, Zahara Meilawaty, Maria Lestari, Izzan M.A. Mazaya

**Affiliations:** a Department of Biomedical Sciences, Faculty of Dentistry, University of Jember, Jember, Indonesia; b Department of Biomedical Sciences, Faculty of Dentistry, University of Jember, Jember, Indonesia

**Keywords:** مستخلص ورقة الكاسافا؛ الكولاجين؛ الخلايا الليفية؛ التهاب اللثة., Cassava leaf extract, Collagen, Fibroblasts, Periodontitis

## Abstract

**Objectives:**

*Porphyromonas gingivalis*, as the main etiology of periodontitis, causes inflammation in the periodontal tissue, which triggers the immune response, fibroblast decline, and collagen destruction, generating attachment loss. Fibroblasts and collagen perform a fundamental role in the repair process of periodontal tissue. This study examined the potential of cassava leaf extract in increasing the quantity of fibroblasts and collagen density in the gingiva of rats with periodontitis.

**Methods:**

A posttest-only control group was used in this study. The experiment involved 24 male Wistar rats divided into four different groups: control group, group induced by *P. gingivalis* and given aquadest, group induced by *P. gingivalis* and given metronidazole, and group induced by *P. gingivalis* and given cassava leaf extract. Gingival tissue was taken after euthanasia, after which histological preparations were made, and fibroblasts and collagen were observed.

**Results:**

One-way analysis of variance revealed that the collagen density and fibroblasts quantity showed a notable difference between each group (p < 0.05), and interestingly, there was no significant difference between metronidazole and cassava leaf extract in the least significant difference test results (p > 0.05).

**Conclusion:**

Cassava leaf extract has the potential to increase fibroblast quantity and collagen density in the gingiva of periodontitis rat models.

## المخلص

**أهداف البحث:**

يمكن أن تثير بكتيريا بورفيروموناس لثوية التي تعتبر العامل الرئيسي لإلتهاب اللثة استجابة مناعية. يمكن أن يقلل الالتهاب عدد الخلايا الليفية ويسبب تدمير الكولاجين، مما يؤدي إلى فقدان التثبيت. للخلايا الليفية والكولاجين دور هام في عملية التئام التهاب اللثة. اختبرت هذه الدراسة إمكانية استخراج ورقة الكاسافا في زيادة عدد الخلايا الليفية وكثافة الكولاجين في لثة فئران مصابة بإلتهاب اللثة.

**طرق البحث:**

كانت هذه دراسة تجريبية مخبرية مع تصميم مجموعة تحكم بعد اختبار وحيد. تم تقسيم 24 فأرا من نوع ويستر ذكورا إلى 4 مجموعات، وهي (1) مجموعة التحكم، (2) المجموعة المستحثة بواسطة بورفيروموناس لثوية والتي تعطى ماء مقطر، (3) المجموعة المستحثة بواسطة بورفيروموناس لثوية والتي تعطى مترونيدازول، (4) المجموعة المستحثة بواسطة بورفيروموناس لثوية والتي تعطى مستخلص ورقة الكاسافا. تم أخذ نسيج اللثة بعد قتل المرحمة، ثم تم إعداد التحضيرات الهستولوجية، وتمت ملاحظة الخلايا الليفية والكولاجين.

**النتائج:**

أظهر اختبار آنوفا ذو المسار الواحد أن عدد الخلايا الليفية وكثافة الكولاجين قد أظهر فرقا كبيرا بين كل مجموعة، وأظهرت نتائج اختبار أقل فرق معنوي أن مترونيدازول ومستخلص ورقة الكاسافا لم يكون لديه فرق كبير.

**الاستنتاجات:**

لدى مستخلص ورقة الكاسافا الإمكانية لزيادة عدد الخلايا الليفية وكثافة الكولاجين في لثة الفئران التي تعاني التهاب دواعم السن.

## Introduction

Periodontitis, which is characterized by chronic periodontal tissue inflammation, including gingival inflammation and alveolar bone loss, is a common dental and oral infection.^1^ According to Basic Health Research (Riskesdas, 2018), periodontitis is one of Indonesia's most prevalent oral health issues with a 74.1% incidence rate.^2^ Numerous species linked to periodontitis, including *Porphyromonas gingivalis*, *Aggregatibacter actinomycetemcomitans*, and *Fusobacterium nucleatum*, as well as polymicrobial aggregates, are able to invade periodontal tissues while evading the host's defense mechanisms.^3^^,^^4^ Periodontal disease can cause damage to the jawbone structure and pain, causing destructive activity. With a more severe level, bacterial infections that continue to grow can even cause death.^3^

Periodontitis is mainly caused by *P. gingivalis*, a type of Gram-negative bacteria that thrives in anaerobic conditions, and is believed to be the primary cause of the inflammatory mechanisms that lead to periodontal disease.^3^^,^^5^^,^^6^ This bacterium produces several virulence factors that cause inflammation in the periodontal tissue, triggering an innate immune response through host receptors, namely toll-like receptor 2 (TLR-2) and TLR-4 on the surface of host cells, causing the host cells to secrete tumor necrosis factor alpha (TNF-α), interleukin-1 (IL-1), IL-6, and IL-8.^7^, ^8^, ^9^ The inflammation can cause a decrease in fibroblast quantity and collagen destruction, resulting in attachment loss and periodontal pocket formation.^10^

Fibroblasts are crucially involved in all three stages of the healing process. They produce a variety of regulatory molecules, which they use to communicate with other populations of the cell involved in healing mechanisms and coordinate the entire repair process. The interaction between fibroblasts and immune cells reveals characteristics of the early phases of wound healing. This suggests that fibroblasts produce the components of the extracellular matrix (ECM) and set up an interaction with endothelial cells and keratinocytes. Finally, by excreting matrix metalloproteinases (MMPs) and other matrix components, fibroblasts contribute to remodeling of the ECM.^11^ During the proliferation phase, fibroblast activity is very crucial.^12^ In this phase, fibroblasts produce collagen (primarily type III), proteoglycans, fibronectin, hyaluronic acid, and other ECM molecules when stimulated by growth factors released by macrophages and other immune cells. MMPs, which break down fibrin clots and encourage cell migration, are also produced by fibroblasts. Coagulation with a new provisional matrix that supports the migration of keratinocyte is required for re-epithelialization.^11^ A healing process is indicated by an increase in the quantity of fibroblasts and collagen density. Factors that slow the healing process are the wound size, location, tissue vascularization, and functional role of the tissue itself.^13^

One of the drugs of choice for periodontitis is metronidazole. Metronidazole is effective in killing *P. gingivalis* but may cause side effects including nausea, xerostomia, impaired sense of taste, stomach cramps, neurotoxicity, headache, ataxia (impaired movement coordination), seizures, and encephalopathy (brain disease).^14^^,^^15^ Therefore, herbal plants may be an alternative to periodontitis therapy due to their negligible side effects.

Natural ingredients that have the potential to be used as an alternative treatment for periodontitis are cassava leaves (*Manihot esculenta* Crantz), which are composed of saponins, tannins, flavonoids, and vitamin C.^16^^,^^17^ They have antibacterial and anti-inflammatory properties.^18^ The flavonoids in cassava leaf extract can reduce pathogenicity, and inhibit the synthesis of nucleic acids, cytoplasmic membrane function, energy metabolism, attachment and biofilm formation, and the porin on the cell membrane. Consequently, the flavonoids are able to inhibit *P. gingivalis* growth.^19^ By inhibiting cyclooxygenase or lipoxygenase as well as leukocyte accumulation, the anti-inflammatory properties of cassava leaf extract can reduce TNF-α expression in periodontitis-affected rat models.^20^^,^^21^ Another study also showed that cassava leaves had effective anti-inflammatory activity at a dose of 179.2 mg/kg body weight (BW) in rats.^22^ Due to its potential in developing new treatments that can enhance the restoration of periodontal structure and function during the healing process of periodontitis, it is important to explore whether cassava leaf extract can increase the number of fibroblasts and density of collagen in the gingival tissue of rats with periodontitis induced by *P. gingivalis*.

## Materials and Methods

### Preparations

This study used an experimental laboratory and a posttest-only control group design. Male Wistar rats weighing 200–250 g and aged 2–3 months made up the study population. Healthy rats had no physical abnormalities, exhibited an active movement response, and had not been used for previous research. Rats were adapted to the premises and food for 1 week prior to intervention. Rats were placed in laboratories with good air circulation under conditions of a 12:12 h light/dark cycle.

### Extraction of cassava leaf

Identification of cassava plants was conducted in the Plant Laboratory, Department of Agricultural Production, State Polytechnic of Jember Indonesia (Jawa Timur, Indonesia). The maceration method was used to make cassava leaf extract. The cassava used was cultivated in Kreongan Jember Indonesia. The cassava leaves chosen were the fifth leaf from the shoot with a weight of 934 g. After being washed, they were cut into small pieces and allowed to air dry for 2 days at room temperature away from the sun. Next, the leaves were oven-dried for 24 h at 400 °C. Then the dried cassava leaves were crushed and sieved to obtain a fine powder of 366 g. Next, maceration was carried out by placing the simplicia in a container or vessel containing a 96% ethanol solution. The ratio of ethanol to simplicia was 6:1 (2.1 L:366 g). The vessel was tightly closed and then stirred every 24 h 150 times clockwise for 3 days, thus allowing the solvent to enter the entire surface of the simplicia. Then the solution was concentrated using a rotary evaporator set to 50 °C and 90 rpm, allowing the semi-solid cassava leaf extract to be obtained.^22^

### Periodontitis rat model

Twenty-four experimental male Wistar rats were divided into four groups of six rats each: a control group (K), a group induced by *P. gingivalis* and given aquadest (K−), a group induced by *P. gingivalis* and given metronidazole (K+), and a group induced by *P. gingivalis* and given cassava leaf extract (KE). The periodontitis rat model was established by inducing *P. gingivalis* in the distobuccal and distolingual left mandibular first molar with a concentration of 12 × 10^9^ CFU/mL as much as 0.05 mL, administered every third day for a half month administered with a tuberculin syringe and a 30-gauge needle. After the rats had periodontitis, they were treated with distilled water, cassava leaf extract at a dose of 179.2 mg/kg BW, and metronidazole at a dose of 2.25 mg/kg BW orally twice a day for 7 days at 08.00 Western Indonesian Time and 18.00 Western Indonesian Time. On the 8th day, euthanasia was carried out and the left lower jaw of the rat was taken.

### Histological examination and image analysis

In this stage, the obtained jaws were decalcified using 10% formic acid for 2 weeks and then followed by dehydration, clearing, and impregnation. Dehydration was conducted by tissue soaking for 60 min using stratified alcohol (70%, 80%, 90%, 100%) to exsiccate water in the tissues. Then the tissue was cleared by immersing it in a solution of xylene. Finally, the impregnation was carried out by wrapping the tissue using filtering paper labeled to avoid mistaken identity of the sample and inserted into the embedding material, namely paraffin at 60 °C for 2 h. Furthermore, block creation (embedding) was carried out, followed by cutting of the tissue planted in paraffin blocks using microtomes 5 μm thick. Then hematoxylin and eosin staining was performed for observation of fibroblasts and Picrosirius Red for collagen density, and the final stage was mounting. Observations and counting of the fibroblast quantity and collagen density were observed with the Olympus CX23 light microscope with 1000× magnification in six different fields of view, and the Camera Microscope Optilab Advance Plus 12.6 MP was used for analysis of the cells. The observation field was along the junctional epithelium. The quantity of fibroblasts was observed by three different observers, whereas the mean collagen density was determined using ImageJ software.

### Statistical analysis

Shapiro–Wilk and Levene tests were employed to decide the normality and homogeneity of the research data. Furthermore, the data underwent analysis through the one-way analysis of variance (ANOVA) test, followed by the post-hoc least significant difference (LSD) test to further examine differences.

## Results

### Histological appearance of fibroblast and Collagen

**Figure 1:**
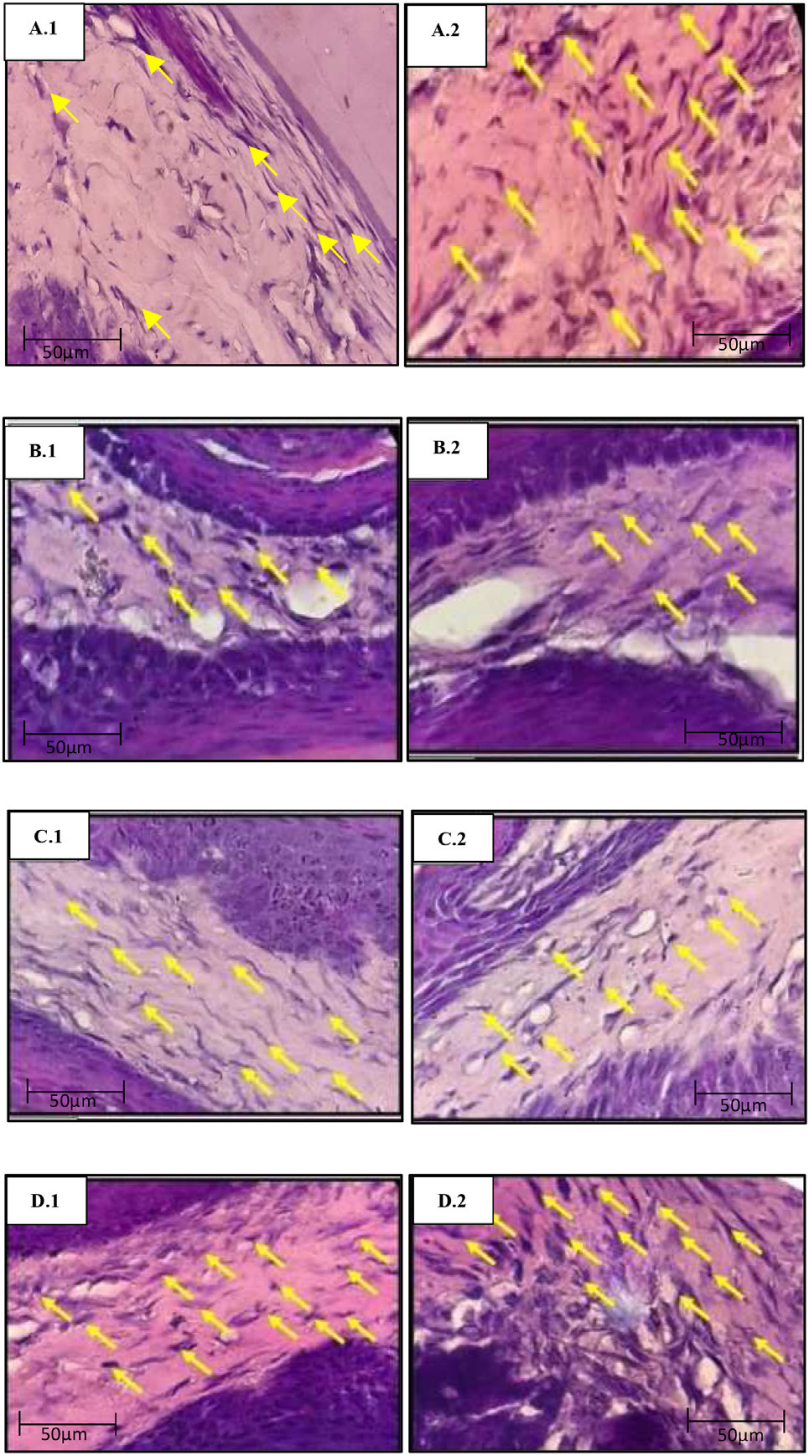
Histological appearance of the rat gingiva with H&E staining (1000 × magnification). (A.1) Buccal gingiva (K); (A.2) lingual gingiva (K); (B.1) buccal gingiva (K−); (B.2) lingual gingiva (K−); (C.1) buccal gingiva (K+); (C.2) lingual gingiva (K+); (D.1) buccal gingiva (EC); (D.2) lingual gingiva (EC). Yellow arrows indicate fibroblast cells.

**Figure 2:**
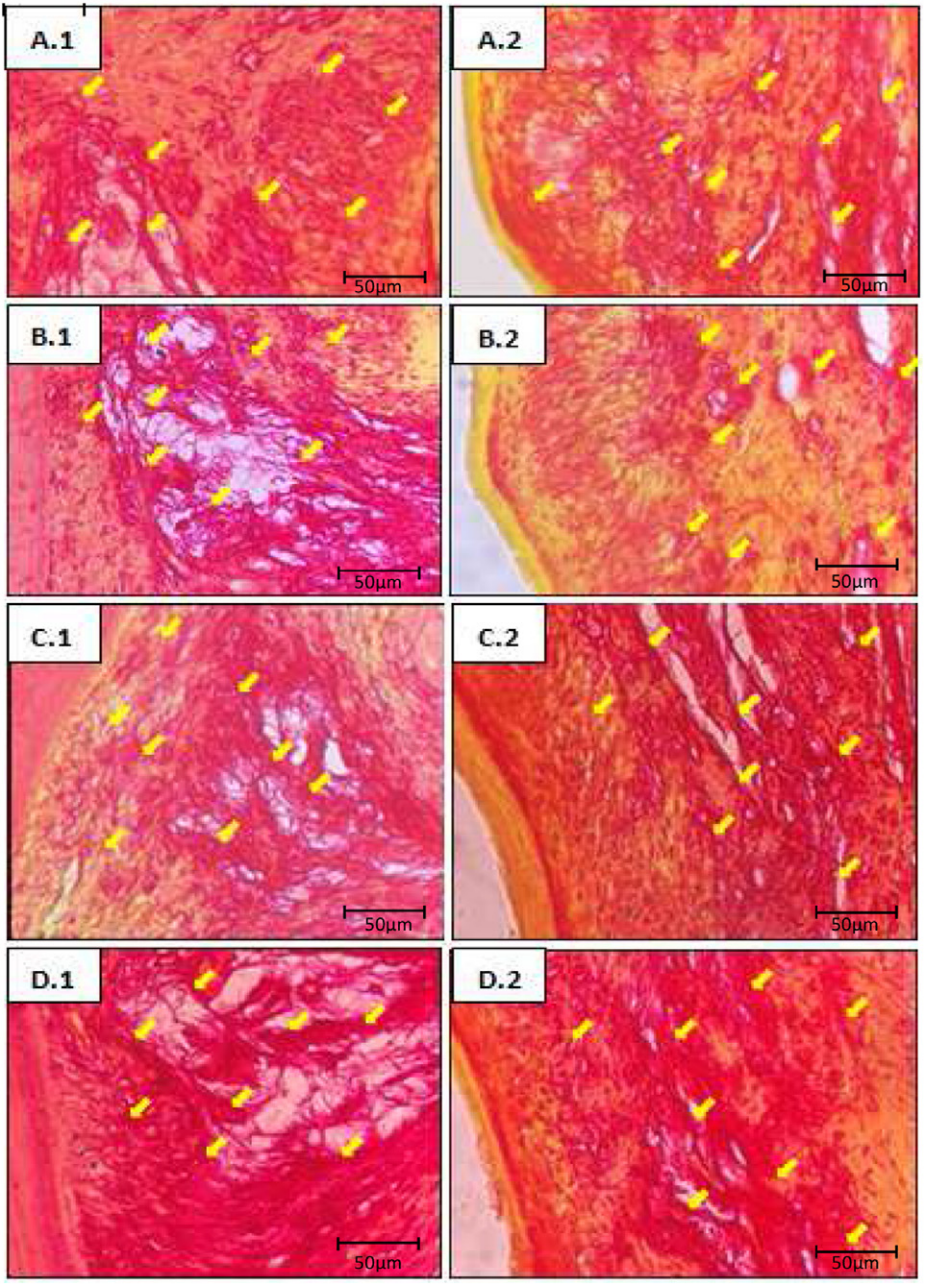
Histological appearance of the rat gingiva with Picrosirius Red staining (1000 × magnification). (A.1) Buccal gingiva (K); (A.2) lingual gingiva (K); (B.1) buccal gingiva (K−); (B.2) lingual gingiva (K−); (C.1) buccal gingiva (K+); (C.2) lingual gingiva (K+); (D.1) buccal gingiva (EC); (D.2) lingual gingiva (EC). Yellow arrows indicate collagen fibers.

**Figure 3:**
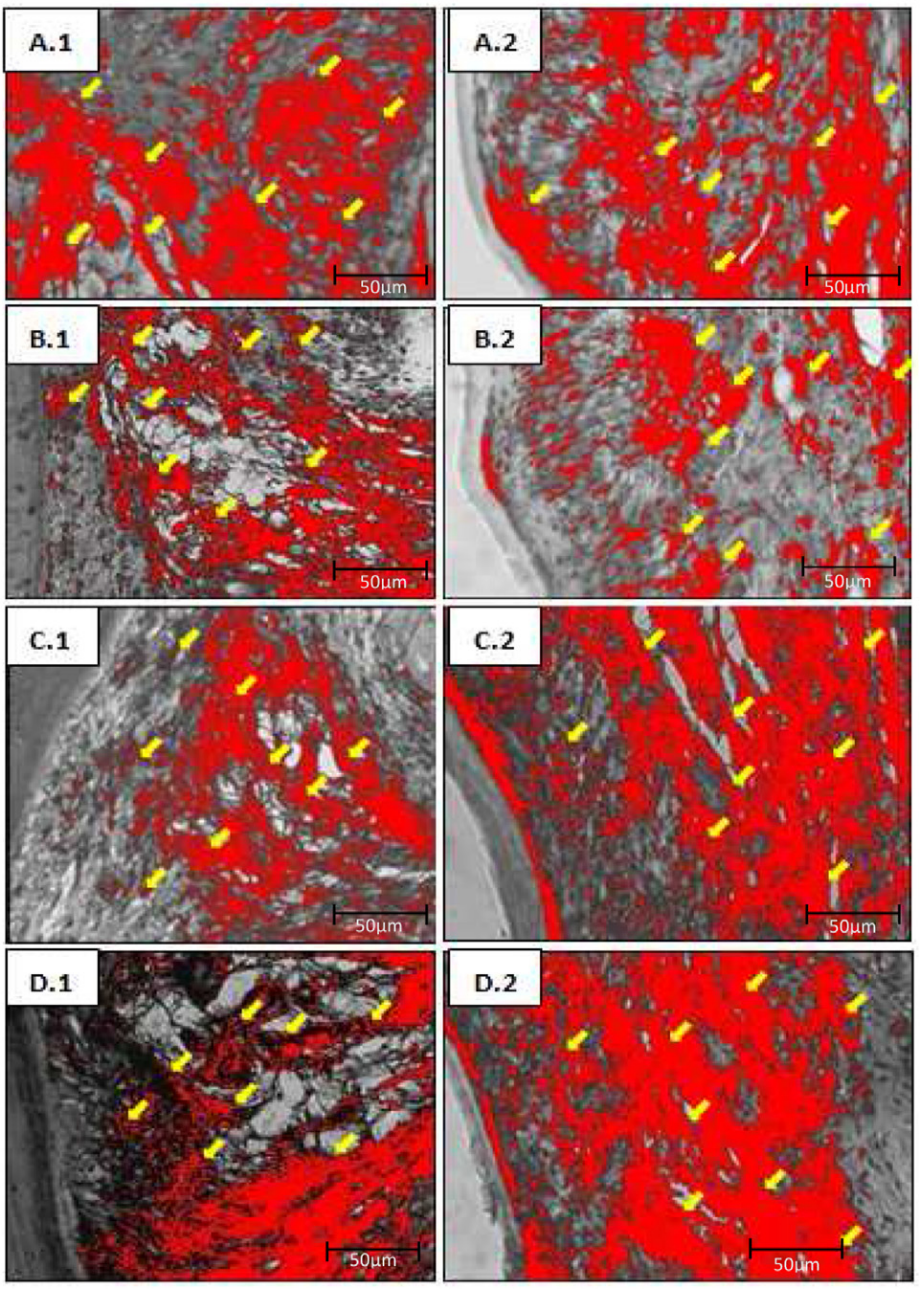
An overview of the collagen density of each group analyzed with ImageJ software. (A.1) Buccal gingiva (K); (A.2) lingual gingiva (K); (B.1) buccal gingiva (K−); (B.2) lingual gingiva (K−); (C.1) buccal gingiva (K+); (C.2) lingual gingiva (K+); (D.1) buccal gingiva (EC); (D.2) lingual gingiva (EC). Yellow arrows indicate collagen density.

H&E staining showed that the fibroblasts had darkly stained cells that appeared dark purple, flat, and branched with oval or elongated cell nuclei (Figure 1). Picrosirius Red staining of the collagen showed red hairs or fibers (Figure 2). The average collagen density was analyzed using ImageJ software, which is shown by the solid red area in Figure 3. The Shapiro–Wilk test revealed that all data exhibited normal distribution, with p > 0.05. Additionally, the Levene test showed that the data were homogeneous, with p > 0.05. The one-way ANOVA test results revealed significant differences between all research groups (p = 0.000).

### Number of fibroblasts

There were more fibroblasts in the control group than in the negative control group. The LSD test results showed a significant difference between the groups (p = 0.000), suggesting that periodontitis due to the induction of *P. gingivalis* can decrease the number of fibroblasts in rats. There were fewer fibroblasts in the negative control group than in the groups administered metronidazole and cassava leaf extract. The LSD test results showed a significant difference (p = 0.000). This suggests that the therapy administered can enhance the fibroblasts quantity in periodontitis rats. The results of the statistical analysis indicated that no significant difference was observed in the quantity of fibroblast in rats given metronidazole and those given cassava leaf extract (p = 0.783). This suggests that the two materials have an equivalent ability to increase the number of fibroblasts in periodontitis rats.

### The density of Collagen

The results of collagen density observations in the control group were higher than those of the negative control group. The outcome of the LSD test indicated a significant difference between the two groups (p = 0.000), suggesting that periodontitis due to the induction of *P. gingivalis* may decrease collagen density in rats. Collagen density in the negative control group was lower than that in the group administered metronidazole and cassava leaf extract. The LSD test results revealed a significant difference (p = 0.000), suggesting that the therapy may have increased collagen density in periodontitis rats. The LSD test results showed no significant difference between collagen density in rats given metronidazole and those given cassava leaf extract (p = 0.821), indicating that both ingredients have the equivalent ability to increase collagen density in periodontitis rats.

## Discussion

**Figure 4:**
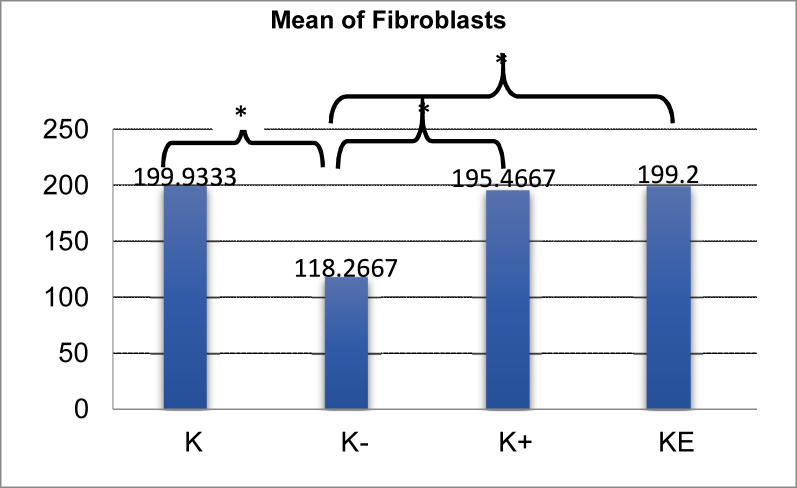
Bar chart showing mean fibroblasts in the rat gingiva of periodontitis model and LSD test results. Information: K = Control group (not induced by *P. gingivalis*). K− = *P. gingivalis* induction rat group + aquadest. K+ = *P. gingivalis* + metronidazole induction rat group. KE = *P. gingivalis* induction rat group + cassava leaf extract (∗p < 0.05).

**Figure 5:**
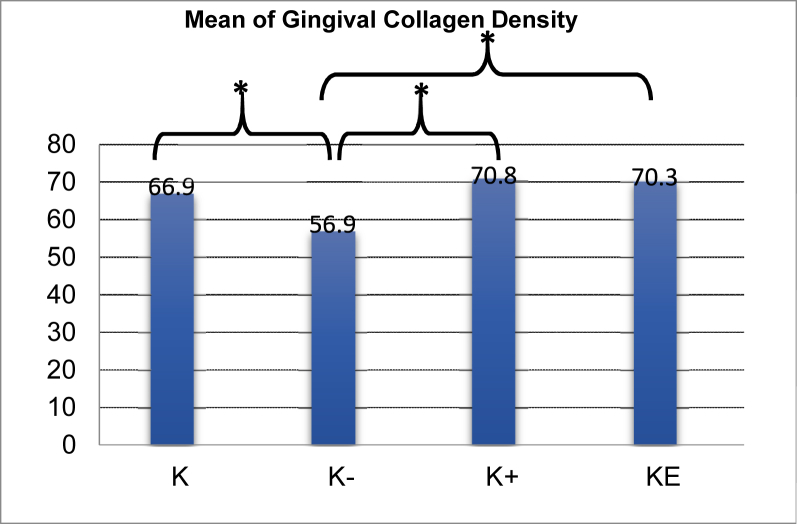
The bar chart presents the mean gingival collagen density of the rat model of periodontitis and the results of the LSD test. Information: K = Control group (not induced by *P. gingivalis*). K− = *P. gingivalis* induction rat group + aquadest. K+ = *P. gingivalis* + metronidazole induction rat group. KE = *P. gingivalis* induction rat group + cassava leaf extract (∗p < 0.05).

The findings of this study demonstrate that cassava leaf extract can increase fibroblast quantity and collagen density in the gingiva of rats with periodontitis following *P. gingivalis* induction. The evidence was shown in the mean number of fibroblasts and collagen density in the gingiva of periodontitis rats treated with cassava leaf extract, which were higher than that in periodontitis rats given metronidazole and treated with aquadest (Figures 4 and 5). In addition, the results of one-way ANOVA demonstrated significant differences in the quantity of fibroblasts and the density of collagen between the negative control group and all other groups. A significant difference was shown by the LSD test results between the periodontitis rat group given cassava leaf extract and the rats given aquadest (Figures 4 and 5).

Rats not induced by *P. gingivalis* showed the highest mean fibroblast quantity of 199.9. The mean indicated a significant difference (p < 0.05) between normal control rats and those induced by *P. gingivalis*, because rats in the normal condition did not experience inflammation. In normal tissue, fibroblasts function to maintain tissue integrity (tissue homeostasis) by regulating ECM turnover. The number can decrease when the tissue is injured.^13^

Periodontitis rats that were given aquadest had a significantly lower number of fibroblasts and collagen density compared to the other groups of rats (p < 0.05). This is because distilled water is neutral and does not have antibacterial or anti-inflammatory properties. Thus, in this group, fibroblast quantity and collagen density decreased due to the injury caused by *P. gingivalis*. Numerous virulence factors produced by *P. gingivalis* either directly or indirectly cause tissue damage and inflammation.^23^ Multiple interconnected molecular pathways including cytokines, growth factors, and MMPs, as well as their regulators and inhibitors, play a role in disease progression.^24^ One of the tissue damage mechanisms in periodontitis is thought to be the ability of pathogens in tooth plaque to induce host cells to release more MMPs.^25^^,^^26^ The primary host factor causing ECM and marginal gingiva collagen breakdown is the MMPs released by local fibroblasts and inflammatory cells. Disintegration of the collagenous fibrous tissue of the marginal gingiva accelerates the resorption of alveolar bone resorption in chronic periodontitis.^27^

Periodontitis rats given metronidazole had a higher number of fibroblasts and collagen density than those given aquadest. This suggests that metronidazole treatment can enhance fibroblast quantity and collagen density. Metronidazole eliminates *P. gingivalis* by inhibiting DNA synthesis and bacterial cell nuclei to prevent colonization and more severe infection. Thus, inflammation can occur in a short time and is followed by tissue regeneration, such as the process of fibroblast proliferation.^28^

Periodontitis rats that were given cassava leaf extract had a significantly different fibroblast quantity and collagen density than those given aquadest (p < 0.05). This means that cassava leaf extract administration can enhance fibroblast quantity and collagen density. This increase is believed to be related to the active chemical compound of cassava leaves such as flavonoids, tannins, saponins, triterpenoids, and vitamin C.^22^ According to Meilawaty and Kusumawardani (2016), cassava leaf extract can reduce expression of the proinflammatory cytokines TNF-α and cyclooxygenase-2 (COX-2).^20^ Another study also showed that cassava leaf extract could reduce MMP-8 expression in rats with ovarian dysfunction with *P. gingivalis*-induced periodontitis. Decreased levels of these cytokines will speed up the healing process and increase fibroblasts quantity and collagen density.^29^ Another study showed that surgically induced skin wounds in rats could be successfully treated with cassava leaf extract. Arranging connective tissue in a parallel pattern to collagen fibers accelerated the remodeling phase by several steps.^30^ Additionally, the *in vitro* wound healing process of cells may be sped up by cassava leaf extract.^31^ Accordingly, Meilawaty et al. (2020) found that cassava leaf extract increased the thickness of the lipopolysaccharide-induced rat gingival epithelium.^32^ In addition, Vicente et al. (2019) showed that cassava leaf extract had the best effects on accelerating healing and tissue repair.^30^

The content of flavonoids in cassava leaves is considered to play a role in increasing the number of fibroblasts and collagen density. Scar tissue development and wound healing both require collagen. Collagen formation develops during the proliferative phase of the wound, which begins on the third day after the physical damage and can extend up to the third week. The next phase is proliferation, which lasts for 3–14 days and is marked by the development of wound granulation. The ECM, in the form of cytokines and fibronectin, stimulate fibroblast cells to multiply. Then proliferating fibroblast cells will move to the wound surface where the fibrin thread previously closed the wound.^33^ By promoting collagen synthesis, reducing macrophages and tissue edema, and boosting fibroblast production, flavonoids promote wound healing.^28^ During the proliferative phase, fibroblast cells gradually form on the surface of the wound and generate fresh collagen fibers. A new collagen fiber will be formed to replace any damaged fibers of collagen that have an uneven shape. The quantity of collagen necessary to heal the wound, however, determines how much collagen is created. The wound surface will be covered with collagen fibers that are reinforced by the presence of fibronectin.^33^ Through a decrease in lipid peroxidation, flavonoids prevent cell necrosis.^34^ By improving their toughness and circulation, lowering cell oxidative stress, and boosting DNA synthesis, lipid peroxidation inhibition can increase the collagen fiber viability.^35^ The anti-inflammatory mechanism of flavonoids is by irreversibly inhibiting the action of COX and lipoxygenase enzymes, which results in the synthesis of inflammatory mediators such as prostaglandins (PGEs), especially decreasing PGE2, prostacyclin, thromboxane, and leukotrienes, thereby affecting the time of the inflammatory process, which speeds up as characterized by the increased proliferation of fibroblasts.^36^

Other ingredients found in cassava leaves that are presumed to play an important role in increasing the number of fibroblasts and collagen density are tannins, which act as antibacterials. Tannins prevent the formation of bacterial cells by inhibiting the reverse transcriptase and DNA topoisomerase enzymes.^37^ Tannins can act as antioxidants and have anti-inflammatory properties that are useful for stopping bleeding, accelerating the healing and inflammation of mucous membranes, and regenerating new tissue.^34^ Tannins can be employed as reactive oxygen species (ROS) scavengers to combat the harmful effects of ROS. Actually, tannins can provide a free radical or ROS an electron, making them more stable substances with fewer adverse effects on the cellular environment.^37^, ^38^, ^39^ Tannins also aid in the scavenging of ROS by boosting antioxidant enzymes when metal ion inactivation generated by free radicals. Numerous tannin-based compounds including proanthocyanidins, ellagic acid-4-O-d-xylopyranoside, gallic acid (3,4,5-trihydroxy benzoic acid), and epigallocatechin gallate have been studied and proven to be highly potent antioxidants.^39^^,^^41^ By causing significant alterations in ribosome pathways, tannins primarily interfere with protein synthesis mechanisms. These changes also affect translation processes in bacterial cells, which ultimately reduces bacterial growth.^40^ It is thought that tannins help speed up the healing of wounds by scavenging free radicals and ROS, encouraging wound contraction, and enhancing capillary vessel growth and fibroblast proliferation.^42^ By inhibiting protein synthesis, altering nucleic acid metabolism, preventing changes to cell wall development, altering cell membrane function, and preventing bacterial growth, tannins demonstrate their antibacterial potential.^43^

Cassava leaves also contain saponins, which have antibacterial, antiviral, antiparasitic, and antifungal activities.^44^ By stimulating the proliferation of fibroblasts and myofibroblast differentiation, saponins can accelerate injury healing. By promoting the synthesis of type I collagen, which is required for wound closure, saponins aid in wound healing.^33^ The ability of the membrane to initiate cell hemolysis is also improved by saponins. When bacteria and saponins combine, bacteria lyse. Saponins can boost monocyte proliferation by increasing macrophage quantity and releasing growth factors that aid in the development of fibroblasts and the synthesis of collagen for the area surrounding the wound. Keratinocytes, which are crucial to the process of wound resurfacing, can move more quickly than saponins.^45^

Triterpenoids in cassava leaves are thought to enhance fibroblast quantity and the density of collagen because they can act as antibacterials, allegedly involving damage to bacterial membranes by lipophilic compounds. Triterpenoids have the ability to interact with porins (transmembrane proteins) on the outer membrane of the bacterial cell wall, causing the formation of robust polymeric bonds that can cause damage to the porin. This reduces the bacterial cell wall's permeability, preventing bacterial cells from receiving nutrients, which in turn prevents bacterial growth or causes the bacteria to die.^46^ Vitamin C in cassava leaves can also kill bacteria by changing the pH of the environment to be unsuitable for bacterial growth (pH < 4). Most bacteria usually grow at a pH of 7–7.5 with a minimum pH of 4.5, so vitamin C can inhibit enzyme activity and bacterial metabolism.^47^^,^^48^

The study also revealed that the fibroblast quantity and density of collagen in periodontitis rats given cassava leaf extract showed no significant difference from periodontitis rats given metronidazole (p < 0.05), which means that cassava leaf extract and metronidazole have the same ability to enhance fibroblast quantity and collagen density. Metronidazole reduces the phagocytosis and ROS generation produced by *P. gingivalis*. When periodontal pathogens such as *P. gingivalis* absorb metronidazole, it binds inexplicably to bacterial DNA, causing DNA damage, impairing DNA function, and ultimately killing the organism.^49^ Meanwhile, cassava leaf extract, which contains flavonoids, tannins, and saponins, can accelerate the inflammation process and accelerate tissue regeneration by increasing the number of fibroblasts.

## Conclusion

In conclusion, our study revealed that Cassava leaf extract (*M. esculenta Crantz*) can enhance fibroblast quantity and collagen density in the rat gingiva after *P. gingivalis* induction. Future studies should explore the potential effects of extended administration of cassava leaf extract (*M. esculenta Crantz*) to fibroblast growth and gingival collagen fibers during the periodontitis healing process by following the mechanism involved in the present study. Developing innovative therapeutic approaches for the repair and regeneration of periodontal tissues is crucial; therefore, the aforementioned findings are of significant importance.

## Source of funding

This research was funded by the University of Jember legalized by Rector Decree of the University of Jember No. 23572/UN25/KP/2022 (Source of funds DIPA-023.17.2.677562.2022; Award Number: 6337/UN25.3.1/LT/2022).

## Conflict of interest

The authors have no conflict of interest to declare.

## Ethical approval

The research protocol was approved by the Ethical Committee of Medical Research Faculty of Dentistry the University of Jember (Reference No. 016/KKEP/FKG-UGM/EC/2022) in 24 January 2022.

## Authors contributions

ADPS: Conceptualization; project administration; methodology; resources; supervision; writing; review and editing. AWSD: Data curation; investigation; writing-original draft. ZM: Formal analysis; software; writing the original draft. ML: Data analysis; writing the original draft. IMAM: Methodology; data analysis; writing the original draft. The final draft of the manuscript's content and similarity index have been reviewed and approved by all authors, who are also accountable for its content. All authors have critically reviewed and approved the final draft and are responsible for the content and similarity index of the manuscript.
